# Impact of Antenatal Corticosteroids on Respiratory and Neurodevelopmental Outcomes in the FGR Rabbit Model

**DOI:** 10.3390/biomedicines14081775

**Published:** 2026-08-06

**Authors:** Katerina Zapletalova, Marnel Greyling, Yannick Regin, Ignacio Valenzuela, Ladislav Krofta, Jan Deprest, Johannes van der Merwe

**Affiliations:** 1 Group Biomedical Sciences, Department of Development and Regeneration, Cluster Woman and Child, KU Leuven, 3000 Leuven, Belgiumhannes.vandermerwe@uzleuven.be (J.v.d.M.); 2 Institute for the Care of Mother and Child, Third Faculty of Medicine, Charles University, 147 00 Prague, Czech Republic; 3 Department of Obstetrics and Gynecology, Division Woman and Child, University Hospitals Leuven, 3000 Leuven, Belgium; 4 Center of Interventional Medicine for Precision and Advanced Cellular Therapy, Universidad de Los Andes, Santiago 7620001, Chile

**Keywords:** fetal growth restriction, antenatal corticosteroids, prenatal treatment, survival, rabbit model

## Abstract

**Background/Objectives**: Antenatal corticosteroids (ACSs) are widely used to improve outcomes in preterm infants, but their effects in the context of fetal growth restriction (FGR) remain incompletely understood. This study investigated the impact of ACSs on pulmonary and neurodevelopmental outcomes using a rabbit mod (K.Z.el) of FGR. **Methods**: FGR was induced at a gestational age (GA) of 25 days (term 31.5 days) by partial uteroplacental vessel ligation (UPVL) in one uterine horn, with the contralateral horn serving as a control. Dams received intramuscular betamethasone (0.1 mg/kg) or saline 24 and 12 h before expected delivery. At GA 30 days, offspring were delivered by caesarean section and allocated to four groups for pulmonary function testing or neurobehavioral assessment on postnatal day 1, followed by histological analyses of the brain, lungs, and placenta. **Results**: ACSs improved respiratory mechanics, particularly static and dynamic compliance, in FGR offspring. PND 1 survival showed a concerning pattern, with numerically lower model-estimated survival in both ACS-exposed groups, although the only statistically significant adjusted comparison was between Control/NoACS and FGR/ACS offspring. Neurobehavioral testing showed lower neurosensory performance after ACS exposure within both control and FGR offspring. Lung morphometry and apoptosis were not significantly altered, while neuronal density and astrogliosis showed isolated regional differences. Placental histology showed a higher junctional zone proportion in untreated FGR placentas than in untreated controls. **Conclusions**: ACSs improved short-term respiratory mechanics without detectable alveolar structural benefit. The observed PND 1 survival pattern and lower neurobehavioral performance warrant cautious interpretation, and these animal findings should not be directly extrapolated to clinical ACS decision-making in FGR pregnancies.

## 1. Introduction

Fetal growth restriction (FGR) is a significant contributor to perinatal mortality and morbidity [1]. The management of FGR is aimed at optimizing the timing of delivery, wherein obstetricians must balance the risk of an ongoing pregnancy in the context of FGR against that of preterm delivery with subsequent prematurity [2,3], frequently resulting in the premature delivery of FGR fetuses. The anticipation of premature delivery offers a strategic window for administering antenatal corticosteroids (ACSs). Maternal administration of a single course of ACSs, such as two doses of 12 mg intramuscular betamethasone administered 24 h apart, reduces respiratory morbidity and short-term complications of prematurity in infants at risk of preterm birth [4]. In extremely preterm infants, ACS exposure has also been associated with reduced neonatal morbidity and mortality [5]. Nevertheless, evidence supporting ACS efficacy specifically in early-onset FGR remains limited, as growth-restricted fetuses have been underrepresented in randomized trial evidence and available data are largely indirect or observational [6].

Furthermore, concerns exist regarding the potential effects of ACSs on other fetal organ systems, particularly the central nervous system, and psychosocial development [7,8].

The rationale for studying ACSs in FGR is complicated by a clinically relevant paradox. Growth-restricted fetuses are often exposed to chronic hypoxemia and endogenous stress, which may accelerate aspects of pulmonary maturation and alter glucocorticoid receptor signaling. Consequently, exogenous ACSs may be less beneficial, or potentially harmful, in compromised fetuses compared with appropriately grown fetuses. This uncertainty is particularly relevant in early-onset FGR, where clinicians must weigh anticipated respiratory benefit against potential neurodevelopmental, metabolic, and survival risks.

Translational research can play a critical role in testing hypotheses encountered through clinical observations. Over the last 15 years, rabbit models of FGR have been extensively characterized. These models are particularly appealing because of their remarkable similarity to humans in terms of hemodichorial placental structure and key organ development [9]. Notably, rabbits exhibit alveolarization in their lungs prior to birth [10] and follow a trajectory of perinatal brain development [11], which enhances their translational potential. Inducing placental underperfusion through uteroplacental vessel ligation (UPVL) results in an FGR phenotype similar to that of human early-onset FGR (EoFGR) associated with high mortality, multiorgan sequelae, and placental histological alterations [12,13,14,15].

In our previous work involving a late preterm rabbit model, exposure to ACSs not only increased neonatal lung function but also led to a sustained growth deficit, which correlated with observable neurobehavioral impairment [16]. In this study, we investigated the short-term neurodevelopmental consequences of a single course of betamethasone within the context of prematurely born, growth-restricted fetuses. We hypothesized that ACSs would improve short-term respiratory function in both FGR and non-FGR offspring, but that this functional benefit would not necessarily be accompanied by structural lung maturation and could be associated with adverse survival or neurobehavioral effects, particularly in FGR offspring.

## 2. Materials and Methods

### 2.1. Animal Model

Time-mated rabbit dams (*New Zealand rabbit*, CEGAV, Chartres, France) were housed individually in cages maintained at 21 °C with 42% humidity and a 12 h light/dark cycle. They had ad libitum access to food and water. The day of conception was considered gestational day (gestational age, GA) 0. Placental underperfusion was surgically induced at GA 25 (full term 31.5 days) via partial uteroplacental vessel ligation (UPVL), following a previously established protocol [17]. The dams were subsequently randomized via research randomizer software (version 4.0, obtained from https://www.randomizer.org/) to receive either 0.1 mg/kg betamethasone (Celestone Chronodose, Schering-Plough Labo N.V., Heist-op-den-Berg, Belgium) or 1 mL of saline intramuscularly (IM) at 24 and 12 h before delivery.

The betamethasone formulation contained betamethasone acetate and betamethasone phosphate and was diluted to a total volume of 1 mL. The selected dose and schedule were based on previously established rabbit ACS protocols [16] and were intended to reproduce the clinically relevant concept of repeated maternal betamethasone exposure shortly before preterm delivery. Because interspecies differences in glucocorticoid pharmacokinetics, metabolism, clearance, and receptor sensitivity preclude a direct mg/kg conversion between rabbits and humans, this regimen should be interpreted as a translationally informed experimental exposure rather than a strict human-equivalent dose.

At GA 30, caesarean section deliveries were performed. Placentas were separated, trimmed from the umbilical cord and membranes, weighed, and fixed in 4% paraformaldehyde (PFA) for 72 h. Euthanasia of dams was performed via intravenous administration of 0.1 mL/kg T61 (Embutramidum 200 mg/Mebezonii iodidum 50 mg/Tetracaini hydrochloridum 5 mg, T61, Boxmeer). Newborn rabbits were numbered and placed in a controlled environment within a heated (34 °C) and humidified (55% relative humidity) incubator (TLC-50 Advance, Brinsea Products, Weston Super Mare, UK). After four hours, survival status was recorded, and the animals were assisted in urination, weighed, and nourished with a commercial milk substitute (Day One, protein 30%, fat 50%; Fox Valley, Lakemoor, IL, USA) enriched with probiotics (Bio-Lapis; Probiotics International, Somerset, UK) and immunoglobulins (Col-o-Cat; SanoBest, Hertogenbosch, The Netherlands).

**Figure 1:**
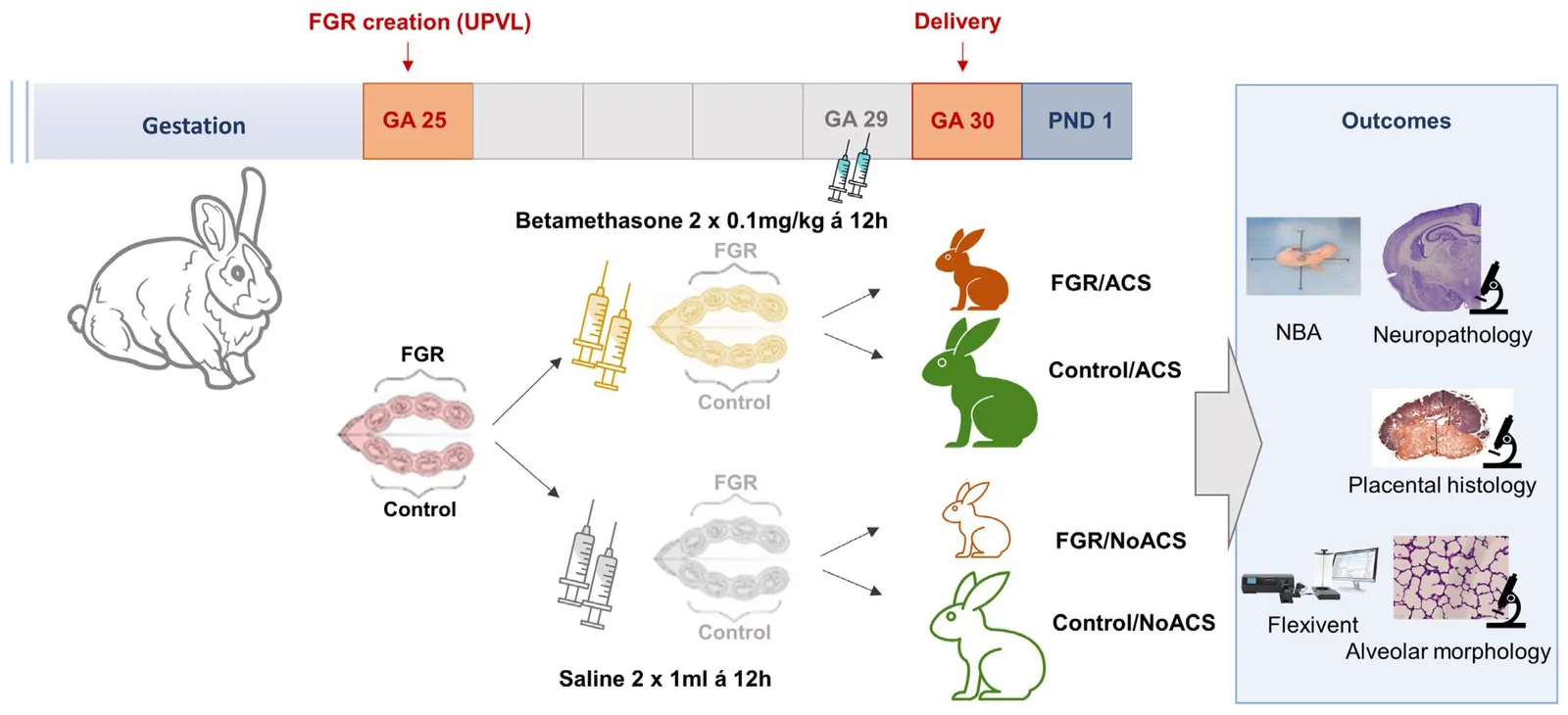
Experimental setup.

On postnatal day 1 (PND 1), litters were allocated for pulmonary function testing or neurobehavioral and neuropathological evaluation. The four experimental groups were FGR with betamethasone (FGR/ACS), FGR without betamethasone (FGR/NoACS), control with betamethasone (Control/ACS), and control without betamethasone (Control/NoACS). The experimental setup is depicted in Figure 1.

### 2.2. UPVL Creation

Anesthesia was induced in pregnant dams via intramuscular ketamine (35 mg/kg Nimatek^®^, Eurovet Animal Health BV, Bladel, The Netherlands) and xylazine (5 mg/kg XYL-M^®^ 2%, VMD, Arendonk, Belgium). Surgical preparations included tocolysis (10 mg/kg medroxyprogesterone, Depo-Provera^®^ SC, Pfizer, Puurs, Belgium) and analgesia (0.03 mg/kg buprenorphine, Vetergesic^®^ SC, Ceva Animal Health, Brussels, Belgium). Anesthesia was maintained through continuous intravenous infusion of ketamine (8–16 mg/kg/h) and xylazine (2.4–4.8 mg/kg/h), with vital signs closely monitored. The surgical UPVL procedure involved the ligation of 33–50% of the vessels supplying each placenta with Vicryl^®^ 5-0 (Ethicon^®^, Johnson & Johnson, Diegem, Belgium), with the contralateral horn used as an internal control. The abdomen was closed using Vicryl^®^ 2-0 and Monocryl^®^ 3-0 (Ethicon^®^, Johnson & Johnson) for the fascia and skin, respectively. The surgical site was infiltrated with levobupivacaine (2 mg/kg Chirocaine^®^, Abbvie, Wavre, Belgium) and aluminum spray (Kela, Hoogstraten, Belgium) for local analgesia.

### 2.3. Placental Histology

Following fixation, placentas were paraffin-embedded and sectioned into 4 μm slides. Slides were stained with cytokeratin lectin and imaged using a Zeiss AxioScan Z1 imaging platform (AxioScan Slide Scanner, Carl Zeiss MicroImaging GmbH, Munich, Germany). Placental zones (decidua, junctional zone, and labyrinth) were manually outlined via QuPath open-source software (version 0.2.0; Belfast, Northern Ireland) [18]. Placental zone volumes were derived from their relative sizes and placental weights via previously established methods [17].

### 2.4. Neurobehavioral Assessment (NBA)

On postnatal day 1 (PND 1), a cohort of offspring underwent a validated NBA protocol [11,19,20,21]. The short-term motor evaluation included assessment of gait, posture, locomotion, head and limb movements, and activity duration. Sensory evaluations included testing of cranial nerves, pain responses, and the righting reflex. All evaluations were captured on video and scored by an independent observer blinded to group allocation. A comprehensive outline of the NBA procedures is provided in the Supplementary Materials.

### 2.5. Brain Collection and Histological Analysis

Following NBA on PND 1, animals received deep sedation via intramuscular ketamine (35 mg/kg) and xylazine (6 mg/kg). This was followed by transcardial perfusion using 0.9% saline with heparin (100 U/mL; 3 min at 30 mL/min), followed by 4% PFA (5 min at 30 mL/min). Brains were extracted and immersion-fixed in 4% PFA for 48 h. After fixation, brain weights were recorded. Brains were paraffin-embedded and sectioned at 4 µm. Three sets of four consecutive coronal sections were taken at 100 µm intervals for two levels, as previously outlined (11): level 1 beginning at the medial septal nucleus and level 2 at the hippocampal formation. Slides were incubated with a mouse monoclonal anti-glial fibrillary acidic protein antibody (GFAP; G6171, Sigma-Aldrich, St. Louis, MO, USA) or processed with terminal deoxynucleotidyl transferase dUTP nick end labeling (TUNEL assay, ApopTag® Fluorescein In Situ Apoptosis Detection Kit, S7110; Millipore, Burlington, MA, USA). Sections were counterstained with Hoechst 33342 (Sigma-Aldrich, Bornem, Belgium). Regions assessed included the frontal cortex (FC), corpus callosum (CC), caudate nucleus (CN), internal capsule (IC), putamen (P), and hippocampal subregions CA1, CA2, and dentate gyrus (DG).

### 2.6. Pulmonary Function Testing

On PND 1, a group of offspring underwent pressure-volume and forced oscillation maneuvers using the FlexiVent system (SciReq; FlexiVent, Montreal, QC, Canada). After sedation with ketamine (35 mg/kg) and xylazine (6 mg/kg), tracheostomy was performed to insert an 18-gauge metal cannula into the trachea. Rabbits were ventilated with a tidal volume of 10 mL/kg and positive end-expiratory pressure of 3 cmH_2_O at 120 breaths/min. Two deep inflation maneuvers were performed prior to PFT to standardize lung volume by inflating the lungs to 30 cmH_2_O. Pressure-volume parameters (inspiratory capacity, static compliance, static elastance, hysteresis) and forced oscillation parameters (tissue damping, tissue elastance, central airway resistance, respiratory system resistance, and dynamic compliance) were determined according to established protocols [22]. The average of three independent measurements for each maneuver, each with a coefficient of determination exceeding 95%, was calculated and treated as a single data point for analysis.

### 2.7. Histological Lung Evaluation

After PFT, a thoracotomy was performed to extract the lungs. A 20-gauge catheter was secured in the trachea, and the left lung was pressure-fixed for 24 h under a constant hydrostatic pressure of 25 cmH_2_O in 4% PFA [22]. Following fixation, the left lung was paraffin-embedded and serially sliced into 5 µm sections. For alveolar morphometry, one slide per lung was stained with hematoxylin and eosin and digitally scanned. Using a semiautomated Fiji plugin (ImageJ version 1.54f, http://fiji.sc/Fiji, accessed on 13 October 2022) with validated capabilities, mean linear intercept (Lm), alveolar air space (Lma), interalveolar septal thickness (Lmw), septal volume density (Vv), and air surface density (Sv) were calculated. Twenty fields per lung were randomly selected in accordance with established stereological principles [10,23].

### 2.8. Statistical Analysis

The sample size calculation was based on previously published data [17,24] showing reduced hippocampal neuronal density in betamethasone-treated rabbits and approximately 10% lower neuronal counts in FGR rabbits on PND 1. These effect sizes informed the estimates when neuronal density was used as the primary outcome. Statistical analyses were performed in R version 4.3.2 using RStudio (RStudio PBC, Boston, MA, USA), primarily with the lme4 and emmeans packages. Graphs were generated in GraphPad Prism version 9.4.1 (GraphPad Software, San Diego, CA, USA).

Because offspring were nested within dams/litters, all analyses accounted for within-litter clustering. Survival at birth and survival on PND 1 were analyzed as separate binary outcomes using generalized linear mixed-effect models fitted with a binomial distribution and logit link. FGR status, ACS treatment, and their interaction were included as fixed effects, and dam was included as a random intercept. Continuous outcomes were analyzed using linear mixed-effect models fitted by restricted maximum likelihood estimation, with the same fixed-effect structure and dam as a random intercept. The general model structures were survival~FGR × Treatment + (1|dam) for survival outcomes and outcome~FGR × Treatment + (1|dam) for continuous outcomes.

Pairwise comparisons among the four experimental groups were based on estimated marginal means and adjusted for multiple comparisons using Tukey’s method. Degrees of freedom for continuous-outcome contrasts were calculated using the Kenward–Roger method. Survival outcomes are reported as model-estimated probabilities with 95% confidence intervals. Continuous outcomes are reported in the text and Supplementary Tables S1–S5 as model-estimated means ± standard error (SE), whereas figures display individual observations summarized using arithmetic means ± standard deviation (SD). Exact effect estimates, test statistics, degrees of freedom, and adjusted *p* values are reported for the comparisons discussed in the text. A two-sided adjusted *p* value < 0.05 was considered statistically significant. For selected secondary outcomes, singular fits occurred because the estimated variance in the dam-level random intercept approached zero. In these cases, the prespecified mixed-effect model was retained to maintain a consistent analytical framework across outcomes; singular fits are disclosed in the Supplementary Materials and the affected secondary outcomes were interpreted cautiously. Per-outcome sample sizes, including the numbers of offspring or placentas and contributing dams in each experimental group, are provided in Supplementary Table S1.

### 2.9. Animal Welfare Monitoring, Refinement, and Humane Endpoints

Animal welfare was monitored throughout the experiment by trained personnel. Animals were checked daily, with particular attention to signs of pain or distress, food and water intake, tremors, locomotor activity, and body weight before procedures and whenever food intake or behavior changed. All potentially painful procedures were performed under general anesthesia. Dams undergoing surgery received general anesthesia combined with long-acting local and systemic analgesia, with clinical monitoring maintained during surgery. Postoperative care included analgesics and antibiotics to minimize pain and reduce infection risk.

Humane endpoints were predefined and assessed daily after surgery in pregnant dams. Endpoint criteria included surgical wound dehiscence or evisceration, abnormal postoperative weight loss, signs of sepsis, severe reduction in spontaneous activity, or other major surgical complications compromising animal welfare.

Neonatal rabbits were assessed at birth, four hours after birth, and on the following morning, and received supportive care including temperature control and assisted feeding when required. Because neonatal survival constituted a study outcome, animals were closely monitored and supportive measures were provided; however, euthanasia was performed if animals developed severe irreversible distress incompatible with survival. Animals meeting predefined humane endpoint criteria were euthanized without delay.

Study protocols were approved by the Ethics Committee for Animal Experimentation of the Faculty of Medicine at KU Leuven (PPL 019/2023; approval date: 28 February 2023), and were performed and reported in accordance with applicable national and European legislation and the ARRIVE 2.0 guidelines [25].

## 3. Results

### 3.1. Survival and Biometrics

This study included offspring from 27 dams, of which 17 received ACSs and 10 received saline. Raw survival at birth was 71/133 (53%) in the FGR/ACS group, 32/76 (42%) in the FGR/NoACS group, 73/108 (68%) in the Control/ACS group, and 50/73 (68%) in the Control/NoACS group. Corresponding survival on PND 1 was 36/133 (27%), 30/76 (39%), 31/108 (28%), and 41/73 (56%), respectively. Raw survival counts and model-estimated biometric outcomes are summarized in Supplementary Table S2.

**Figure 2:**
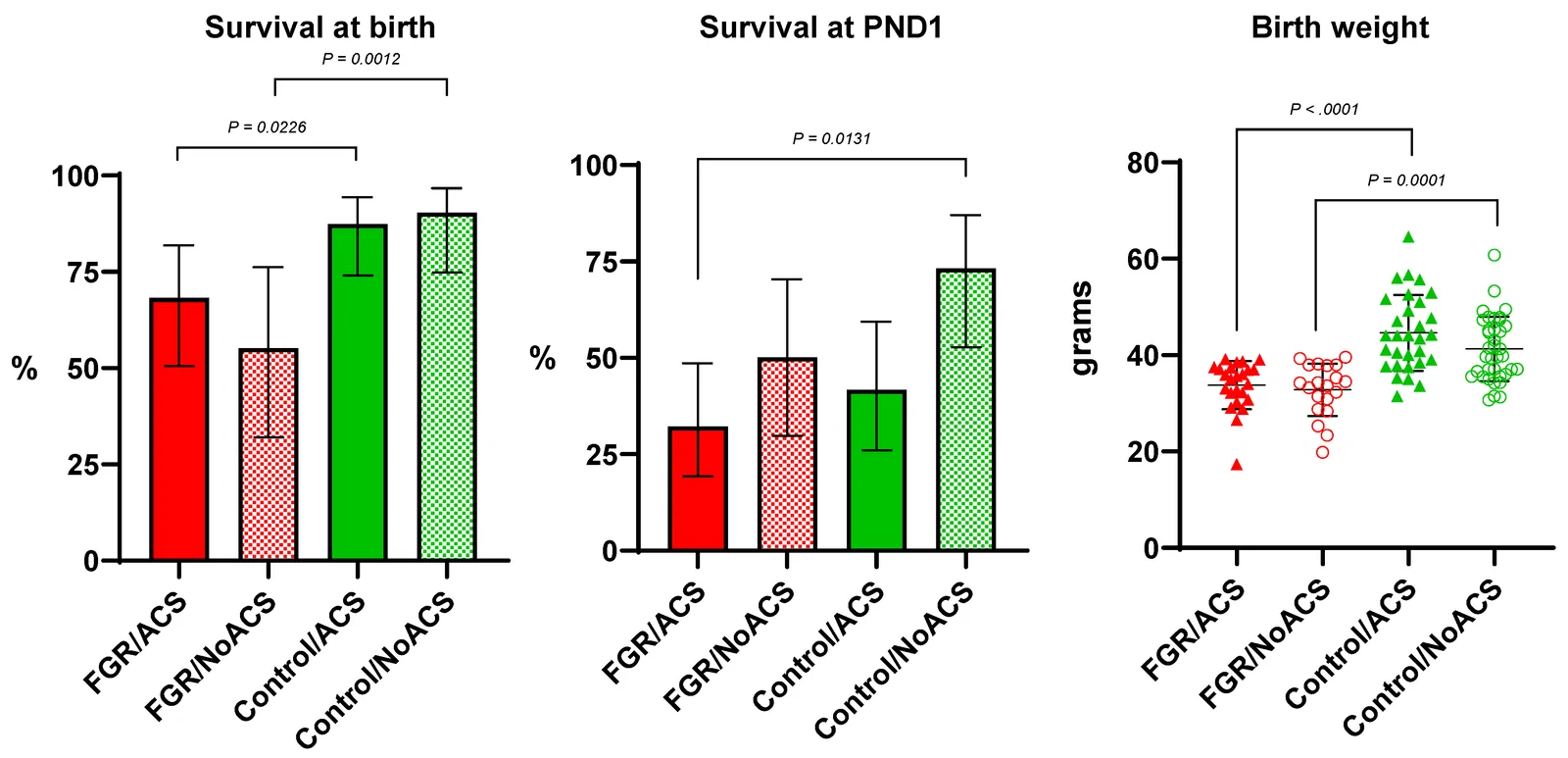
Survival and birth weight of newborn rabbits according to FGR status and antenatal corticosteroid exposure. Model-estimated survival probabilities at birth and on postnatal day 1 (PND 1) are presented with 95% confidence intervals. Birth weight is shown as individual offspring values, with horizontal lines and error bars indicating mean ± SD. Survival outcomes were analyzed using separate binomial generalized linear mixed-effect models, and birth weight using a linear mixed-effect model, with dam included as a random intercept to account for within-litter clustering. Pairwise comparisons were based on estimated marginal means and adjusted for multiple comparisons using Tukey’s method. Exact adjusted *p* values are shown for the selected statistically significant pairwise comparisons displayed in the figure. FGR, fetal growth restriction; ACS, antenatal corticosteroids; NoACS, no antenatal corticosteroids; SD, standard deviation.

In the binomial mixed-effect model, survival at birth was lower in FGR than in control offspring within both treatment strata. At birth, the odds of survival were higher in Control/NoACS than in FGR/NoACS offspring (OR for Control/NoACS vs. FGR/NoACS = 7.66, 95% CI 1.87–31.39, adjusted *p* = 0.0012) and higher in Control/ACS than in FGR/ACS offspring (OR for Control/ACS vs. FGR/ACS = 3.23, 95% CI 1.12–9.27, adjusted *p* = 0.0226). On PND 1, the odds of survival were higher in Control/NoACS than in FGR/ACS offspring (OR for Control/NoACS vs. FGR/ACS = 5.75, 95% CI 1.31–25.37, adjusted *p* = 0.0131). The odds of survival were numerically higher in Control/NoACS than in Control/ACS offspring, but this comparison did not remain significant after Tukey adjustment (OR for Control/NoACS vs. Control/ACS = 3.82, 95% CI 0.85–17.12, adjusted *p* = 0.0991). This observation was therefore interpreted cautiously as a mortality signal rather than evidence of a statistically confirmed control-specific ACS effect. Model-estimated survival probabilities with 95% confidence intervals are presented in Figure 2.

Birth weight was lower in FGR offspring than in the corresponding control groups. FGR/NoACS offspring weighed less than Control/NoACS offspring (estimated difference = 8.60 g, SE = 1.86, adjusted *p* = 0.0001), and FGR/ACS offspring weighed less than Control/ACS offspring (estimated difference = 10.89 g, SE = 1.75, adjusted *p* < 0.0001). ACS exposure did not significantly affect birth weight within the FGR groups (estimated difference = −1.38 g, SE = 2.24, adjusted *p* = 0.9261). The fetal-to-placental weight ratio did not differ significantly between groups after Tukey adjustment.

### 3.2. Neurobehavioral and Neuropathological Findings

#### 3.2.1. Neurobehavioral Findings

Neurobehavioral assessment was performed on PND 1 in 43 offspring from 11 dams, including 12 FGR/ACS offspring from 7 dams, 9 FGR/NoACS offspring from 2 dams, 12 Control/ACS offspring from 5 dams, and 10 Control/NoACS offspring from 3 dams.

Neuromotor scores were lower in FGR than in control offspring within both treatment groups. FGR/NoACS offspring had lower scores than Control/NoACS offspring by 3.91 points (adjusted *p* = 0.0022), whereas FGR/ACS offspring had lower scores than Control/ACS offspring by 6.22 points (adjusted *p* < 0.0001). Although neuromotor scores were numerically lower in FGR/ACS than in FGR/NoACS offspring, this difference did not remain statistically significant after Tukey adjustment (estimated difference = 4.44 points, adjusted *p* = 0.0743).

**Figure 3:**
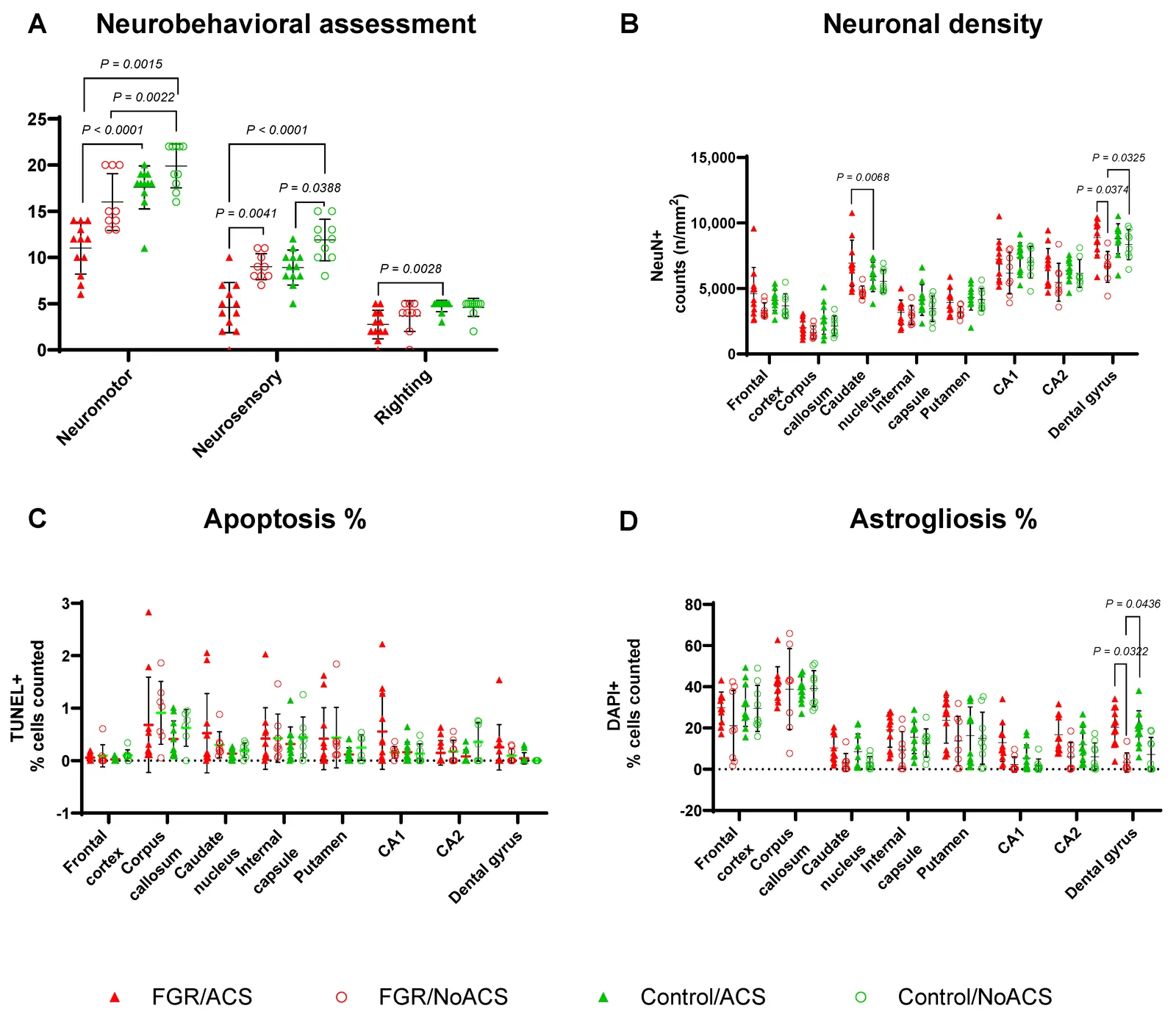
Neurobehavioral and neuropathological outcomes at postnatal day 1. (**A**) Neurobehavioral assessment included neuromotor score, neurosensory score, and righting reflex. (**B**) Neuronal density was evaluated across the frontal cortex, corpus callosum, caudate nucleus, internal capsule, putamen, CA1, CA2, and dentate gyrus. (**C**) Apoptosis and (**D**) astrogliosis were assessed across the same predefined brain regions. Individual symbols represent individual measurements, and data are summarized as mean ± SD. Exact Tukey-adjusted *p* values derived from linear mixed-effect models accounting for clustering within dams are shown for selected statistically significant pairwise comparisons. No statistically significant pairwise differences were detected for apoptosis. FGR, fetal growth restriction; ACS, antenatal corticosteroids; NoACS, no antenatal corticosteroids; SD, standard deviation.

Neurosensory scores were reduced by both FGR and ACS exposure. Within the untreated groups, scores were lower in FGR/NoACS than in Control/NoACS offspring by 2.90 points (adjusted *p* = 0.0307). Within the ACS-treated groups, scores were lower in FGR/ACS than in Control/ACS offspring by 4.33 points (adjusted *p* = 0.0001). ACS exposure was also associated with lower neurosensory scores within both control and FGR offspring, with estimated differences of 2.98 points between Control/NoACS and Control/ACS offspring (adjusted *p* = 0.0388) and 4.42 points between FGR/NoACS and FGR/ACS offspring (adjusted *p* = 0.0041). Righting reflex scores were lower in FGR/ACS than in Control/ACS offspring by 1.97 points (adjusted *p* = 0.0028); no other pairwise comparisons were statistically significant. Neurobehavioral outcomes at PND 1 are shown in Figure 3A. Selected Tukey-adjusted pairwise comparisons for neurobehavioral and neuropathological outcomes are provided in Supplementary Table S3.

#### 3.2.2. Neuropathological Findings

Brain histological analyses included offspring from 11 dams. Depending on tissue availability and staining quality, neuronal density was evaluated in 36–40 offspring, apoptosis in 34–40 offspring, and astrogliosis in 40–41 offspring across the examined brain regions.

No statistically significant differences in neuronal density were detected in most brain regions. In the caudate nucleus, neuronal density was higher in FGR/ACS than in Control/ACS offspring by 1771 cells/mm^2^ (adjusted *p* = 0.0068). In the dentate gyrus, neuronal density was lower in FGR/NoACS than in Control/NoACS offspring by 1711 cells/mm^2^ (adjusted *p* = 0.0325) and lower than in FGR/ACS offspring by 2095 cells/mm^2^ (adjusted *p* = 0.0374).

Apoptosis did not differ significantly between groups in any examined brain region after Tukey adjustment. Astrogliosis also showed no statistically significant group differences in most regions. In the dentate gyrus, however, astrogliosis was lower in FGR/NoACS than in Control/ACS and FGR/ACS offspring, with estimated differences of 14.86 and 15.21, respectively (adjusted *p* = 0.0436 and 0.0322). Neuropathological outcomes are shown in Figure 3B–D.

### 3.3. Pulmonary Function and Structure

#### 3.3.1. Pulmonary Function

Pulmonary function was assessed on PND 1 in up to 70 offspring from 15 dams; central airway resistance, tissue damping, and tissue elastance were available for 69 offspring. Model-estimated group values for the reported pulmonary function parameters are summarized in Supplementary Table S4. Inspiratory capacity was higher in Control/ACS than in Control/NoACS offspring (estimated difference = 5.91 mL/kg, adjusted *p* = 0.0179) and FGR/NoACS offspring (estimated difference = 6.61 mL/kg, adjusted *p* = 0.0184). Within FGR offspring, inspiratory capacity was numerically higher after ACS exposure, but the difference did not reach statistical significance after Tukey adjustment (estimated difference = 5.54 mL/kg, adjusted *p* = 0.0726).

**Figure 4:**
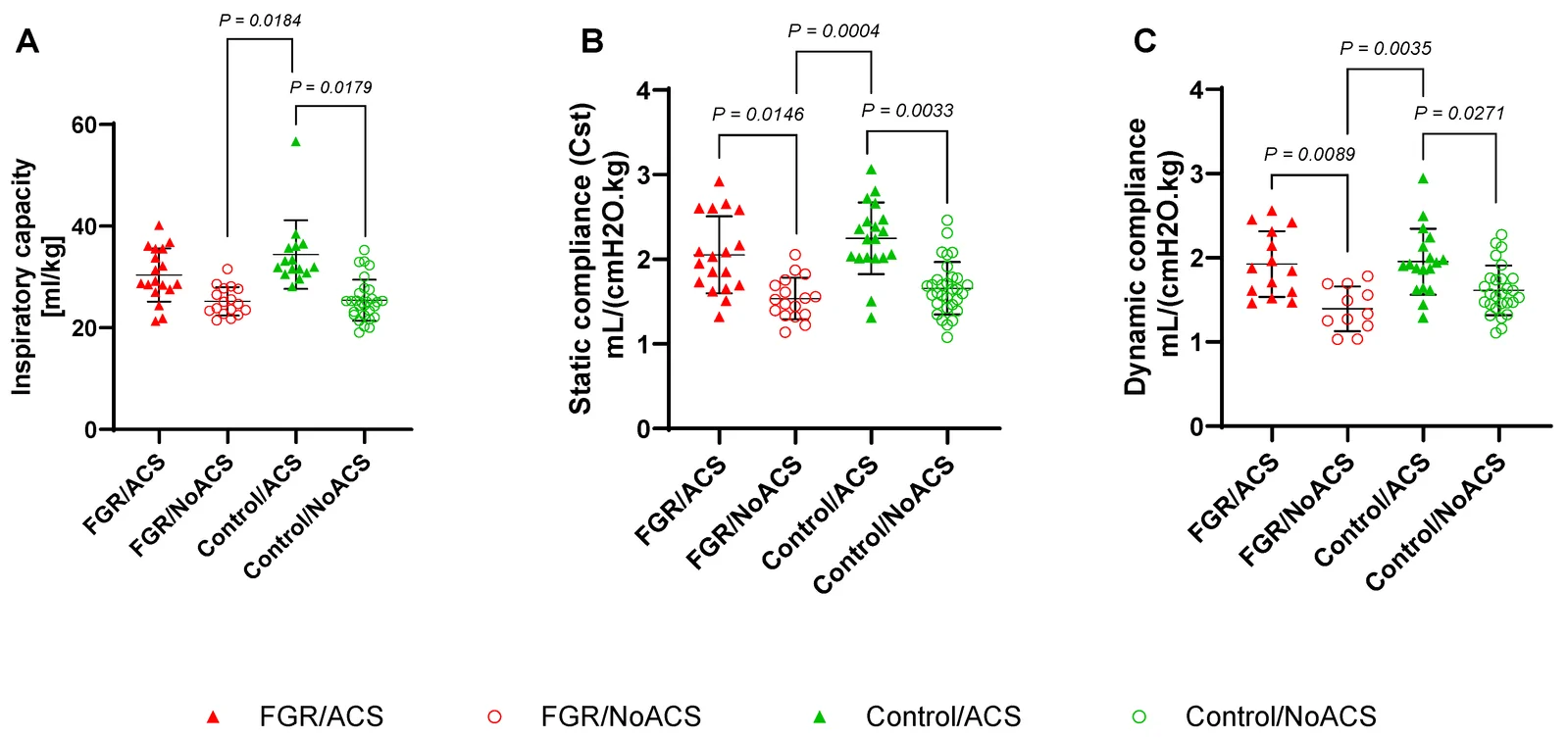
Pulmonary function at postnatal day 1. (**A**) Inspiratory capacity, (**B**) static compliance at 25 cmH_2_O, and (**C**) dynamic compliance. Individual symbols represent measurements from individual offspring, and data are summarized as means ± SD. Statistical comparisons were performed using linear mixed-effect models with FGR status, ACS treatment, and their interaction as fixed effects and dam as a random intercept, followed by Tukey-adjusted pairwise comparisons. Exact adjusted *p* values are shown for statistically significant pairwise comparisons. FGR, fetal growth restriction; ACS, antenatal corticosteroids; NoACS, no antenatal corticosteroids; SD, standard deviation.

Static compliance (Cst) was higher in Control/ACS than in Control/NoACS offspring (estimated difference = 0.562 mL/[cmH_2_O·kg], adjusted *p* = 0.0033) and FGR/NoACS offspring (estimated difference = 0.766 mL/[cmH_2_O·kg], adjusted *p* = 0.0004). Within FGR offspring, static compliance was higher after ACS exposure (estimated difference = 0.560 mL/[cmH_2_O·kg], adjusted *p* = 0.0146). Dynamic compliance showed a similar pattern: values were higher in Control/ACS than in Control/NoACS offspring (estimated difference = 0.355 mL/[cmH_2_O·kg], adjusted *p* = 0.0271) and FGR/NoACS offspring (estimated difference = 0.595 mL/[cmH_2_O·kg], adjusted *p* = 0.0035), and higher in FGR/ACS than in FGR/NoACS offspring (estimated difference = 0.559 mL/[cmH_2_O·kg], adjusted *p* = 0.0089) (Figure 4).

Among the additional respiratory mechanical parameters, FGR/ACS offspring had lower respiratory system resistance (estimated difference = 0.218 cmH_2_O·s/mL, adjusted *p* = 0.0153), tissue damping (estimated difference = 1.364 cmH_2_O/mL, adjusted *p* = 0.0094), tissue elastance (estimated difference = 5.022 cmH_2_O/mL, adjusted *p* = 0.0129), and respiratory system elastance (estimated difference = 9.50 cmH_2_O/mL, adjusted *p* = 0.0031) than FGR/NoACS offspring. Conversely, hysteresis was higher in FGR/ACS than in FGR/NoACS offspring (estimated difference = 0.312 mL·cmH_2_O, adjusted *p* = 0.0283). Central airway resistance did not differ significantly between groups. Complete Tukey-adjusted pairwise comparisons for the reported pulmonary function outcomes are provided in Supplementary Table S5.

#### 3.3.2. Lung Morphometry

**Figure 5:**
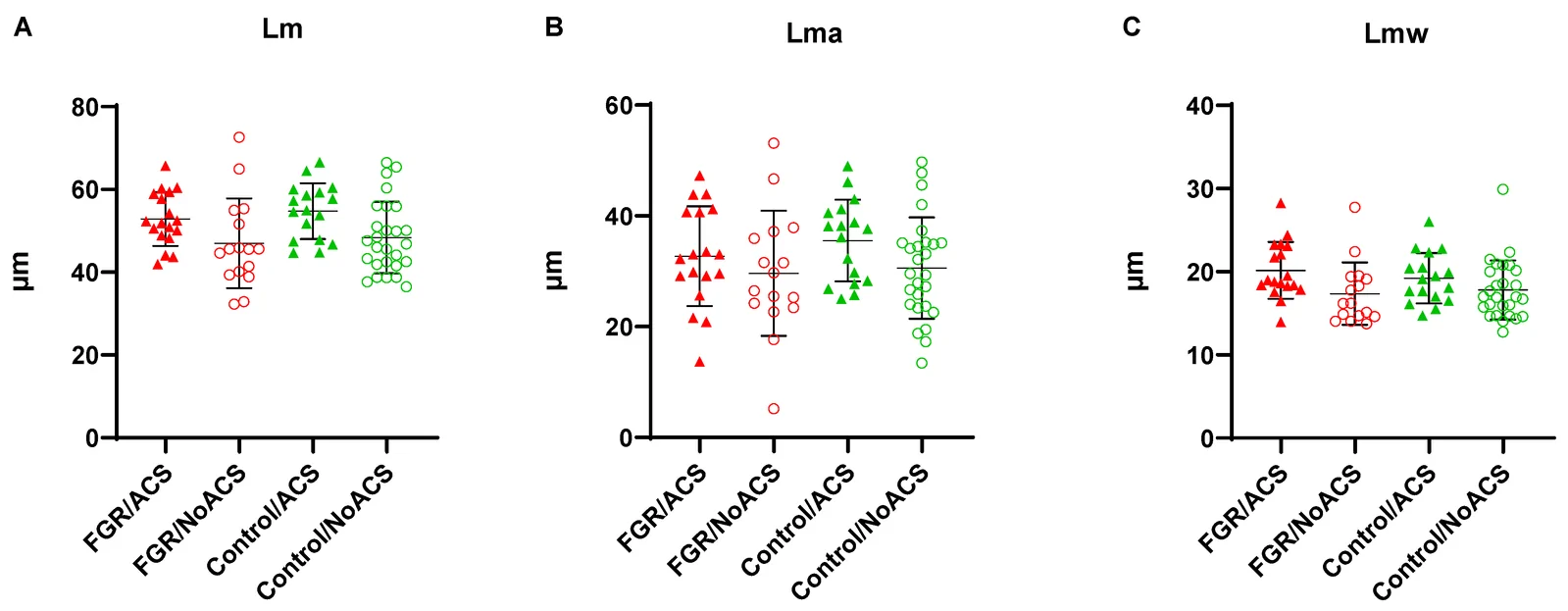
Alveolar morphometry at postnatal day 1. (**A**) Mean linear intercept (Lm), (**B**) alveolar airspace size (Lma), and (**C**) interalveolar septal thickness (Lmw). Individual symbols represent measurements from individual offspring, and data are summarized as means ± SD. Statistical comparisons were performed using linear mixed-effect models with FGR status, ACS treatment, and their interaction as fixed effects and dam as a random intercept, followed by Tukey-adjusted pairwise comparisons. No statistically significant pairwise differences were detected after adjustment. FGR, fetal growth restriction; ACS, antenatal corticosteroids; NoACS, no antenatal corticosteroids; SD, standard deviation.

Alveolar morphometry was assessed in 65 offspring from 15 dams. No statistically significant pairwise differences were detected in mean linear intercept (Lm), alveolar airspace size (Lma), interalveolar septal thickness (Lmw), septal volume density (Vv), or air surface density (Sv) after Tukey adjustment. The smallest adjusted *p* values were 0.2346 for Lm, 0.4897 for Lma, 0.5705 for Lmw, 0.8323 for Vv, and 0.4614 for Sv. Thus, the observed differences in pulmonary mechanics were not accompanied by detectable differences in alveolar structure on PND 1 (Figure 5).

### 3.4. Placental Histology

Placental histology was assessed in 100 placentas from 25 dams, including 22 FGR/ACS placentas from 11 dams, 19 FGR/NoACS placentas from 6 dams, 24 Control/ACS placentas from 11 dams, and 35 Control/NoACS placentas from 10 dams. This asymmetry may reflect a higher rate of fetal or placental loss on the ligated side before delivery in untreated FGR pregnancies and should be considered when interpreting both placental and survival outcomes. Total placental area did not differ significantly between groups after Tukey adjustment (all adjusted *p* ≥ 0.2564). Similarly, no statistically significant pairwise differences were detected in the decidual zone proportion (all adjusted *p* ≥ 0.1596) or labyrinth zone proportion (all adjusted *p* ≥ 0.2865).

**Figure 6:**
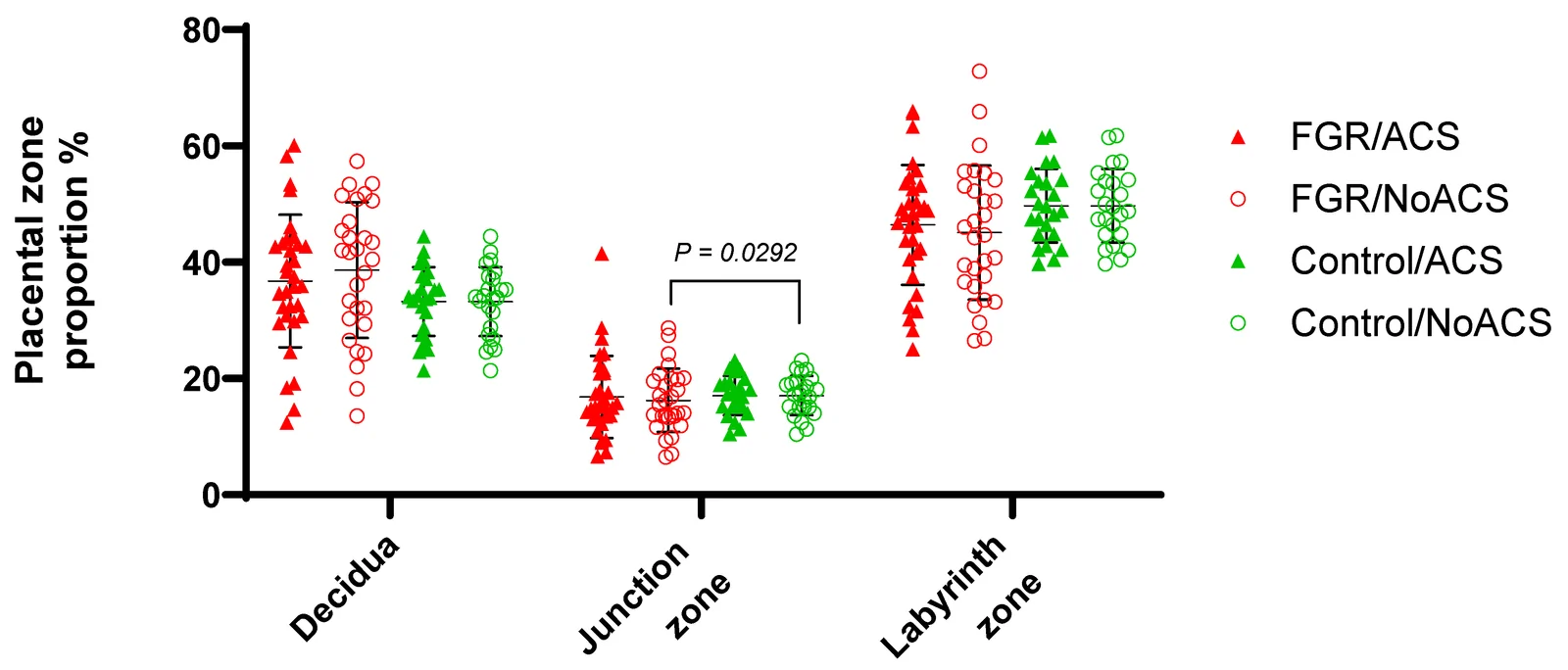
Placental zone proportions. Decidual, junctional, and labyrinth zone proportions were calculated relative to the total placental area. Individual symbols represent measurements from individual placentas, and data are summarized as arithmetic means ± SD. Statistical comparisons were performed using linear mixed-effect models including FGR status, ACS treatment, and their interaction as fixed effects and dam as a random intercept, followed by Tukey-adjusted pairwise comparisons. The junctional zone proportion was higher in FGR/NoACS than in Control/NoACS placentas (estimated difference = 3.992 percentage points, adjusted *p* = 0.0292). No statistically significant pairwise differences were detected in the decidual or labyrinth zone proportions. FGR, fetal growth restriction; ACS, antenatal corticosteroids; NoACS, no antenatal corticosteroids; SD, standard deviation.

The junctional zone proportion was higher in FGR/NoACS than in Control/NoACS placentas (estimated difference = 3.992 percentage points, SE = 1.41, df = 82.6, *t* = 2.831, adjusted *p* = 0.0292). No statistically significant difference was detected between FGR/ACS and Control/ACS placentas (Control/ACS − FGR/ACS estimated difference = 0.626 percentage points, adjusted *p* = 0.9715) or between FGR/NoACS and FGR/ACS placentas (estimated difference = −0.353 percentage points, adjusted *p* = 0.9987). Thus, the only statistically significant pairwise difference in placental zone proportions was observed between untreated FGR and untreated control placentas. Placental zone proportions are shown in Figure 6.

## 4. Discussion

In this study, we investigated the effects of antenatal corticosteroids (ACSs) in a rabbit model of fetal growth restriction (FGR) induced by placental underperfusion. The principal finding was that ACSs improved short-term pulmonary mechanics in FGR offspring, particularly static and dynamic compliance. These functional benefits were not accompanied by detectable differences in alveolar morphometry on postnatal day 1 (PND 1). Neurobehavioral performance also differed across groups, with the lowest neurosensory scores observed in FGR/ACS offspring. PND 1 survival showed a concerning pattern, with numerically lower model-estimated survival in both ACS-exposed groups; however, the only statistically significant comparison was between Control/NoACS and FGR/ACS offspring. Taken together, these findings indicate an acute respiratory functional benefit of ACSs in preterm FGR rabbits that was not paralleled by detectable structural lung maturation and occurred alongside short-term neurobehavioral and survival concerns.

The pulmonary findings are consistent with the established role of ACSs in promoting preterm respiratory adaptation, but they also demonstrate a functional-structural disconnect. Previous studies in guinea pig and sheep models of FGR have shown that ACSs can improve indices of pulmonary maturation, including lung compliance and surfactant-related outcomes, and may promote selected aspects of structural lung development [26,27]. In the present study, ACS exposure increased static and dynamic compliance in FGR offspring and was accompanied by favorable differences in several secondary respiratory mechanical parameters. Inspiratory capacity was numerically higher in FGR/ACS than in FGR/NoACS offspring, but this difference did not remain statistically significant after Tukey adjustment. Moreover, no statistically significant differences were detected in mean linear intercept, alveolar airspace, interalveolar septal thickness, septal volume density, or air surface density. The short-term functional benefit may therefore have been mediated by surfactant-related effects, lung-fluid clearance, or changes in tissue mechanics rather than by measurable structural remodeling. Because surfactant protein expression, alveolar epithelial maturation, and lung-fluid clearance were not assessed, this mechanistic interpretation remains speculative.

The neurodevelopmental findings likewise require cautious interpretation. FGR/ACS offspring had lower neuromotor scores than both control groups, a lower righting reflex score than Control/ACS offspring, and the lowest neurosensory scores. Control/ACS and FGR/NoACS offspring also had lower neurosensory scores than Control/NoACS offspring. In contrast, apoptosis did not differ significantly between groups, while neuronal density and astrogliosis showed only isolated regional differences. Thus, the neurobehavioral findings were not accompanied by a consistent pattern of overt neuropathological injury. This functional-histological dissociation may reflect subtle alterations in synaptic function, connectivity, myelination, neuroactive steroid signaling, neurotransmitter systems, or stress responsiveness that are not captured by neuronal density, TUNEL staining, or GFAP quantification. Previous studies in FGR animal models have similarly reported region- and timing-dependent neurodevelopmental effects of ACSs, including alterations in neuroactive steroid synthesis and white matter development [28,29,30]. The present results therefore do not demonstrate structural neurotoxicity, but neither do they establish neurodevelopmental safety beyond the short observation period.

The survival findings are the most clinically sensitive aspect of this study. At birth, survival was lower in FGR than in control offspring within both treatment strata, consistent with the severity of the experimental FGR phenotype. On PND 1, however, the only statistically significant Tukey-adjusted comparison was between Control/NoACS and FGR/ACS offspring. Although model-estimated survival was numerically lower in Control/ACS than in Control/NoACS offspring, this comparison did not reach statistical significance after adjustment. The direct comparison between FGR/ACS and FGR/NoACS offspring was also not statistically significant. The present data therefore do not demonstrate either a control-specific detrimental effect of ACSs or an adverse effect confined exclusively to FGR offspring. Rather, they raise the possibility of broader early postnatal vulnerability after ACS exposure in this rabbit model, while the wide confidence intervals and limited number of contributing dams warrant caution. Potential explanations include species-specific glucocorticoid sensitivity, developmental timing, metabolic stress, immune modulation, cardiovascular instability, caesarean delivery order, or neonatal care factors that could not be fully separated within the present design.

The survival pattern contrasts with the recent systematic review and meta-analysis by van de Meent et al., which did not identify evidence that ACSs increased mortality specifically in FGR animal models and reported beneficial effects on fetal lung maturation [31]. Differences between that evidence base and the present findings may reflect species, gestational timing, corticosteroid regimen, severity of placental insufficiency, neonatal care, and timing of outcome assessment. Clinical data also generally support respiratory benefits of ACSs. In extremely preterm infants, ACS exposure has been associated with improved survival and neurodevelopmental outcomes (5), while the ALPS trial demonstrated reduced short-term respiratory morbidity among infants at risk of late-preterm delivery, although neonatal hypoglycemia was more frequent after betamethasone exposure [32]. However, these human populations differ substantially from a model of early-onset FGR with severe placental insufficiency. The current findings should therefore be considered hypothesis-generating and should not be translated directly into clinical ACS decision-making in FGR pregnancies.

Placental histology showed a higher junctional zone proportion in untreated FGR placentas than in untreated controls, whereas no corresponding difference was detected between the ACS-exposed groups. Total placental area and the decidual and labyrinth zone proportions did not differ significantly between groups. Importantly, the direct comparison between FGR/NoACS and FGR/ACS placentas was not statistically significant. These findings should therefore not be interpreted as evidence that ACSs normalized placental structure; rather, they indicate an isolated difference between untreated FGR and untreated control placentas. The asymmetric number of contributing dams in the NoACS placental cohort, with fewer dams represented in FGR/NoACS than in Control/NoACS, further suggests that preferential fetal or placental loss on the ligated side may have influenced the available placental sample. This limitation is directly relevant to interpretation of both placental morphology and survival.

### Strengths and Limitations

A major strength of this study is the use of a well-established rabbit model of early-onset FGR with controlled ACS administration, enabling simultaneous assessment of survival, respiratory function, neurobehavior, brain histology, lung morphometry, and placental histology. The paired uterine-horn design provided FGR and non-ligated control offspring within the same dams, while the use of mixed-effect models accounted for clustering of offspring within dams/litters. Randomized maternal treatment, blinded neurobehavioral scoring, explicit per-outcome sample-size reporting, and presentation of effect estimates and Tukey-adjusted pairwise comparisons further strengthen the transparency of the analysis.

Several limitations must nevertheless be emphasized. First, follow-up was limited to PND 1, preventing determination of whether the neurobehavioral findings were transient, whether survival differences persisted, or whether structural brain or lung changes emerged later. Second, rabbits may have heightened glucocorticoid sensitivity, and the developmental timing of the perinatal brain trajectory differs across species, limiting direct extrapolation to human pregnancies [11,33,34,35]. Potential interspecies differences in glucocorticoid receptor expression and sensitivity were not measured and remain one possible explanation for the observed pattern. Third, although the ACS regimen followed established rabbit protocols, maternal and fetal betamethasone concentrations were not measured. Therefore, the assumption that this dosing regimen mimics repeated maternal ACS exposure remains a theoretical premise of the experimental design rather than a confirmed biological state in this rabbit model. Species-specific differences in metabolism, clearance, receptor sensitivity, and developmental timing may therefore have contributed to the results. Fourth, survival was assessed at predefined early time points rather than by exact time-to-event recording. Consequently, the mortality signal is based on a single early postnatal endpoint and may have been influenced by neonatal care, caesarean delivery order, litter-level factors, or unmeasured physiological variables. Fifth, the proposed mechanisms, including surfactant-mediated effects, altered lung-fluid clearance, oxidative stress, angiogenic changes, and altered glucocorticoid signaling, were not directly investigated. Finally, the estimated dam-level random-intercept variance was zero in several secondary respiratory models, resulting in singular fits; these supportive outcomes should therefore be interpreted cautiously. Future studies should incorporate longer follow-up, exact time-to-event recording, pharmacokinetic measurements, mechanistic endpoints, and cross-species comparisons of glucocorticoid exposure and receptor sensitivity in rabbits, sheep, and humans.

## 5. Conclusions

ACS administration in this rabbit model of FGR improved short-term respiratory mechanics, particularly static and dynamic compliance, but was not associated with detectable differences in alveolar morphometry. Neurobehavioral performance was lowest in FGR/ACS offspring for several outcomes, whereas brain histology did not show a consistent pattern of structural injury. The PND 1 survival pattern, including numerically lower model-estimated survival in ACS-exposed controls that did not remain statistically significant after Tukey adjustment, requires cautious interpretation and should not be extrapolated directly to human FGR pregnancies. These findings highlight the complex balance between acute respiratory functional benefit and potential short-term neurobehavioral and survival concerns after ACS exposure in compromised pregnancies. Longer follow-up, mechanistic investigation, pharmacokinetic assessment, and cross-species validation are required to determine which fetuses are most likely to benefit from ACSs and whether additional monitoring or adjunctive interventions may be warranted in early-onset FGR.

## Data Availability

The original contributions presented in this study are included in the article/Supplementary Materials. Further inquiries can be directed to the corresponding author.

## Supplementary Materials

The following supporting information can be downloaded at: https://www.mdpi.com/article/10.3390/biomedicines14081775/s1, Neurobehavioral assessment; Table S1: Per-outcome sample sizes and contributing dams; Table S2: Survival and biometric outcomes; Table S3: Selected pairwise comparisons of neurobehavioral and neuropathological outcomes; Table S4: Pulmonary function parameters at postnatal day 1; Table S5: Tukey-adjusted pairwise comparisons of pulmonary function outcomes at postnatal day 1.
