# Supplementary material for: Impact of Antenatal Corticosteroids on Respiratory and Neurodevelopmental Outcomes in the FGR Rabbit Model
Source: Biomedicines. 2026 Aug 6;14(8):1775. doi: 10.3390/biomedicines14081775 (PMC13509963; doi:10.3390/biomedicines14081775)
Supplement: Supplementary file 1 [file biomedicines-14-01775-s001.zip › biomedicines-4385067-supplementary.pdf]

Supplementary material

Neurobehavioral assessment

Neurobehavioral PND1 evaluation based on a modification of neurobehavioral scoring protocol previously described.[1] For each animal, testing is videotaped and scored by a blinded observer. The kits are evaluated in a designated space close to their pen with auditory and olfactory contamination kept to a minimum. Before handling they remain undisturbed in this assessment area for a 3-5 min. adaptation period.

- Cranial nerves are assessed by testing smell (olfaction is tested by recording time to aversive response to a cotton swab soaked with pure ethanol), sucking, and swallowing (by introduction of formula into the kits' mouth with a plastic syringe), and head turn to feeding. The responses are graded on a scale of 0 to 3, 0 being the worst response and 3 the best response.
- Motor examination includes tone, motor activity, and locomotion on a flat surface, righting reflex, and gait. The righting reflex is assessed when the kits are placed on their backs and the number of times turned prone (within 2s) from supine position in 5 tries is registered. Gait is examined based on a modification by Georgiadis et al.[2]Locomotion is assessed as described by Kannan et al. [3]

Sensory examination is limited to touch on the face (touching the face with cotton swab on both sides) and extremities as well as pain on limbs (mild pin prick).

Table S1. Per-outcome sample sizes and contributing dams.

Outcome	FGR/ACS	FGR/NoACS	Control/ACS	Control/NoACS	Total
**Neurobehavioral assessment**	**12 / 7**	**9 / 2**	**12 / 5**	**10 / 3**	**43 / 11**
**Neuronal density**[1]	**12 / 7**	**8 / 2**	**12 / 5**	**8 / 3**	**up to 40 / 11**
**Brain apoptosis**[1]	**12 / 7**	**8 / 2**	**12 / 5**	**8 / 3**	**up to 40 / 11**
**Brain astrogliosis**[1]	**12 / 7**	**8 / 2**	**12 / 5**	**9 / 3**	**up to 41 / 11**
**Pulmonary function testing**[2]	**12 / 5**	**11 / 4**	**18 / 7**	**27 / 7**	**up to 70 / 15**
**Lung morphometry**	**12 / 5**	**11 / 4**	**16 / 7**	**26 / 7**	**65 / 15**
**Placental histology**	**22 / 11**	**19 / 6**	**24 / 11**	**35 / 10**	**100 / 25**

Note. Values are shown as n offspring or placentas / n dams. Dam counts across groups do not sum to the total because the same dam could contribute both FGR and control offspring or placentas. [1]Sample sizes varied according to brain region and tissue or staining availability: neuronal density, n = 36-40; apoptosis, n = 34-40; and astrogliosis, n = 40-41. [2]Pulmonary function testing included up to 70 offspring; tissue damping, tissue elastance,

and central airway resistance were available for 69 offspring. Group-specific values indicate the maximum number available for the respective outcome.

Table S2. Survival and biometric outcomes.

The study included offspring from 27 dams, of which 17 received antenatal corticosteroids (ACS) and 10 received saline (NoACS).

Outcome	FGR/ACS	FGR/NoACS	Control/ACS	Control/NoACS
Survival at birth	71/133 (53%)	32/76 (42%)	73/108 (68%)	50/73 (68%)
Survival on PND1	36/133 (27%)	30/76 (39%)	31/108 (28%)	41/73 (56%)
Birth weight, g	34.032 ± 2.519[a]	32.648 ± 1.820[b]	44.917 ± 1.805	41.245 ± 1.238
FPWR	6.9388 ± 0.5262	6.9083 ± 0.3812	7.5672 ± 0.6305	7.0964 ± 0.4657

Survival values are presented as the raw number of surviving offspring/total number of offspring (%). Birth weight and fetal-to-placental weight ratio are presented as model-estimated means ± SE. Survival outcomes were analyzed using separate binomial generalized linear mixed-effects models, whereas birth weight and fetal-to-placental weight ratio were analyzed using linear mixed-effects models. All models included FGR status, ACS treatment, and their interaction as fixed effects and dam as a random intercept. Pairwise comparisons were based on estimated marginal means and adjusted using Tukey's method. [a]Adjusted $P < 0.0001$ versus Control/ACS; [b]adjusted $P = 0.0001$ versus Control/NoACS. FGR, fetal growth restriction; ACS, antenatal corticosteroids; NoACS, no antenatal corticosteroids; PND1, postnatal day 1; FPWR, fetal-to-placental weight ratio; SE, standard error.

Table S3. Selected pairwise comparisons of neurobehavioral and neuropathological outcomes

Outcome / brain region	Contrast (first group − second group)	Estimated difference	SE	df	*t* ratio	Adjusted *P*
Neuromotor score	Control/NoACS − FGR/NoACS	3.91	0.994	33.13	3.933	**0.0022**
Neuromotor score	Control/NoACS − FGR/ACS	8.35	1.505	9.29	5.549	**0.0015**
Neuromotor score	Control/ACS − FGR/ACS	6.22	0.937	36.71	6.635	**<0.0001**
Neuromotor score	FGR/NoACS − FGR/ACS	4.44	1.585	10.12	2.804	0.0743
Neurosensory score	Control/NoACS − Control/ACS	2.983	0.961	12.28	3.104	**0.0388**
Neurosensory score	Control/NoACS − FGR/NoACS	2.900	1.001	36.72	2.897	**0.0307**
Neurosensory score	Control/NoACS − FGR/ACS	7.317	0.952	14.97	7.689	**<0.0001**
Neurosensory score	Control/ACS − FGR/ACS	4.333	0.904	38.91	4.793	**0.0001**
Neurosensory score	FGR/NoACS − FGR/ACS	4.417	0.995	11.70	4.439	**0.0041**
Righting reflex	Control/ACS − FGR/ACS	1.969	0.520	38.60	3.786	**0.0028**
Neuronal density, caudate nucleus	Control/ACS − FGR/ACS	−1771	500	30.38	−3.539	**0.0068**
Neuronal density, dentate gyrus	Control/NoACS − FGR/NoACS	1710.8	590	31.06	2.901	**0.0325**
Neuronal density, dentate gyrus	FGR/NoACS − FGR/ACS	−2094.9	645	9.90	−3.248	**0.0374**
Apoptosis, caudate nucleus	Control/ACS − FGR/ACS	−0.423	0.174	29.89	−2.423	0.0943
Astrogliosis, dentate gyrus	Control/ACS − FGR/NoACS	14.86	4.76	10.40	3.124	**0.0436**
Astrogliosis, dentate gyrus	FGR/NoACS − FGR/ACS	−15.21	4.64	10.90	−3.279	**0.0322**

Note. Estimated differences, standard errors, degrees of freedom, t ratios, and adjusted P values were derived from linear mixed-effects models including FGR status, ACS treatment, and their interaction as fixed effects and dam as a random intercept. Degrees of freedom were calculated using the Kenward–Roger method. Pairwise comparisons were based on estimated marginal means and adjusted for multiple comparisons using Tukey's method. The estimated difference represents the first group minus the second group listed in each contrast. Units correspond to those used for the respective outcome. FGR, fetal growth restriction; ACS, antenatal corticosteroids; NoACS, no antenatal corticosteroids; SE, standard error; df, degrees of freedom.

Table S4. Pulmonary function parameters at postnatal day 1.

Parameter	FGR/ACS	FGR/NoACS	Control/ACS	Control/NoACS
Inspiratory capacity, mL/kg	30.5011 ± 1.4112	24.9588 ± 1.5789[a]	31.5672 ± 1.2774	25.6570 ± 1.1590[a]
Static compliance (Cst), mL/(cmH_2O·kg)	2.0482 ± 0.1105[e]	1.4879 ± 0.1250[b]	2.2539 ± 0.0990	1.6917 ± 0.0876[b]
Dynamic compliance, mL/(cmH_2O·kg)	1.9273 ± 0.0968[e]	1.3681 ± 0.1113[b]	1.9626 ± 0.0855	1.6080 ± 0.0713[a]
Respiratory system resistance, cmH_2O·s/mL	0.2869 ± 0.0372[e]	0.5048 ± 0.0419[bc]	0.2582 ± 0.0328	0.3560 ± 0.0268
Respiratory system elastance, cmH_2O/mL	14.9945 ± 1.3169[e]	24.4903 ± 1.4854[bd]	12.9605 ± 1.1618	16.0535 ± 0.9481
Central airway resistance, cmH_2O·s/mL	0.0369 ± 0.0240	0.1071 ± 0.0270	0.0382 ± 0.0217	0.0984 ± 0.0197
Tissue damping (G), cmH_2O/mL	2.6105 ± 0.2157[e]	3.9742 ± 0.2434[bd]	2.3876 ± 0.1902	2.6850 ± 0.1583
Tissue elastance (H), cmH_2O/mL	10.1803 ± 0.8310[e]	15.2018 ± 0.9377[bd]	9.2131 ± 0.7326	9.7028 ± 0.6098
Hysteresis (A), mL·cmH_2O	1.1198 ± 0.0666[ae]	0.8075 ± 0.0759[bc]	1.3439 ± 0.0594	1.0417 ± 0.0517[b]

Note. Data are presented as model-estimated means ± SE derived from linear mixed-effects models including FGR status, ACS treatment, and their interaction as fixed effects and dam as a random intercept. Pairwise comparisons were based on estimated marginal means and adjusted for multiple comparisons using Tukey's method. [a] Adjusted $P < 0.05$ versus Control/ACS; [b] adjusted $P < 0.01$ versus Control/ACS; [c] adjusted $P < 0.05$ versus Control/NoACS; [d] adjusted $P < 0.01$ versus Control/NoACS; [e] adjusted $P < 0.05$ versus FGR/NoACS. Pulmonary function testing included up to 70 offspring from 15 dams; central airway resistance, tissue damping, and tissue elastance were available for 69 offspring. FGR, fetal growth restriction; ACS, antenatal corticosteroids; NoACS, no antenatal corticosteroids; SE, standard error.

Table S5. Tukey-adjusted pairwise comparisons of pulmonary function outcomes at postnatal day 1.

Outcome	Contrast (first group - second group)	Estimated difference	SE	df	t ratio	Adjusted P
Inspiratory capacity, mL/kg	Control/NoACS - Control/ACS	-5.910	1.74	15.5	-3.399	**0.0179**
Inspiratory capacity, mL/kg	Control/NoACS - FGR/NoACS	0.698	1.48	62.7	0.472	0.9649
Inspiratory capacity, mL/kg	Control/NoACS - FGR/ACS	-4.844	1.85	17.6	-2.617	0.0759
Inspiratory capacity, mL/kg	Control/ACS - FGR/NoACS	6.608	2.06	24.5	3.201	**0.0184**
Inspiratory capacity, mL/kg	Control/ACS - FGR/ACS	1.066	1.37	61.5	0.779	0.8635
Inspiratory capacity, mL/kg	FGR/NoACS - FGR/ACS	-5.542	2.16	26.3	-2.566	0.0726
Static compliance (Cst), mL/(cmH_2O·kg)	Control/NoACS - Control/ACS	-0.562	0.134	16.2	-4.208	**0.0033**
Static compliance (Cst), mL/(cmH_2O·kg)	Control/NoACS - FGR/NoACS	0.204	0.125	64.8	1.625	0.3720
Static compliance (Cst), mL/(cmH_2O·kg)	Control/NoACS - FGR/ACS	-0.357	0.143	18.2	-2.487	0.0956
Static compliance (Cst), mL/(cmH_2O·kg)	Control/ACS - FGR/NoACS	0.766	0.163	25.5	4.692	**0.0004**
Static compliance (Cst), mL/(cmH_2O·kg)	Control/ACS - FGR/ACS	0.206	0.116	62.4	1.778	0.2935
Static compliance (Cst), mL/(cmH_2O·kg)	FGR/NoACS - FGR/ACS	-0.560	0.171	26.9	-3.271	**0.0146**
Dynamic compliance, mL/(cmH_2O·kg)	Control/NoACS - Control/ACS	-0.3546	0.114	18.4	-3.120	**0.0271**
Dynamic compliance, mL/(cmH_2O·kg)	Control/NoACS - FGR/NoACS	0.2399	0.132	60.3	1.812	0.2779
Dynamic compliance, mL/(cmH_2O·kg)	Control/NoACS - FGR/ACS	-0.3194	0.124	19.6	-2.584	0.0776
Dynamic compliance, mL/(cmH_2O·kg)	Control/ACS - FGR/NoACS	0.5945	0.150	21.6	3.968	**0.0035**
Dynamic compliance, mL/(cmH_2O·kg)	Control/ACS - FGR/ACS	0.0353	0.121	64.5	0.290	0.9914
Dynamic compliance, mL/(cmH_2O·kg)	FGR/NoACS - FGR/ACS	-0.5592	0.157	22.1	-3.551	**0.0089**
Respiratory system resistance, cmH_2O·s/mL	Control/NoACS - Control/ACS	0.0978	0.0435	19.6	2.245	0.1458
Respiratory system resistance, cmH_2O·s/mL	Control/NoACS - FGR/NoACS	-0.1488	0.0553	42.9	-2.688	**0.0482**
Respiratory system resistance, cmH_2O·s/mL	Control/NoACS - FGR/ACS	0.0691	0.0475	19.9	1.454	0.4822
Respiratory system resistance, cmH_2O·s/mL	Control/ACS - FGR/NoACS	-0.2465	0.0597	14.9	-4.132	**0.0044**
Respiratory system resistance, cmH_2O·s/mL	Control/ACS - FGR/ACS	-0.0287	0.0506	65.4	-0.566	0.9417
Respiratory system resistance, cmH_2O·s/mL	FGR/NoACS - FGR/ACS	0.2179	0.0626	15.5	3.479	**0.0153**
Respiratory system elastance, cmH_2O/mL	Control/NoACS - Control/ACS	3.09	1.54	19.6	2.006	0.2197
Respiratory system elastance, cmH_2O/mL	Control/NoACS - FGR/NoACS	-8.44	1.96	42.9	-4.305	**0.0005**
Respiratory system elastance, cmH_2O/mL	Control/NoACS - FGR/ACS	1.06	1.68	19.9	0.629	0.9214
Respiratory system elastance, cmH_2O/mL	Control/ACS - FGR/NoACS	-11.53	2.11	14.9	-5.457	**0.0004**
Respiratory system elastance, cmH_2O/mL	Control/ACS - FGR/ACS	-2.03	1.79	65.4	-1.135	0.6695
Respiratory system elastance, cmH_2O/mL	FGR/NoACS - FGR/ACS	9.50	2.22	15.5	4.282	**0.0031**
Central airway resistance, cmH_2O·s/mL	Control/NoACS - Control/ACS	0.0602	0.0296	15.7	2.036	0.2172
Central airway resistance, cmH_2O·s/mL	Control/NoACS - FGR/NoACS	-0.0087	0.0261	62.6	-0.334	0.9871
Central airway resistance, cmH_2O·s/mL	Control/NoACS - FGR/ACS	0.0615	0.0316	17.8	1.949	0.2439
Central airway resistance, cmH_2O·s/mL	Control/ACS - FGR/NoACS	-0.0689	0.0353	24.6	-1.953	0.2328
Central airway resistance, cmH_2O·s/mL	Control/ACS - FGR/ACS	0.0013	0.0239	60.8	0.054	0.9999
Central airway resistance, cmH_2O·s/mL	FGR/NoACS - FGR/ACS	0.0702	0.0369	26.2	1.900	0.2524

Outcome	Contrast (first group - second group)	Estimated difference	SE	df	t ratio	Adjusted P
Tissue damping (G), cmH_2O/mL	Control/NoACS - Control/ACS	0.2974	0.255	19.2	1.166	0.6547
Tissue damping (G), cmH_2O/mL	Control/NoACS - FGR/NoACS	-1.2892	0.323	42.6	-3.993	**0.0014**
Tissue damping (G), cmH_2O/mL	Control/NoACS - FGR/ACS	0.0745	0.278	19.5	0.268	0.9931
Tissue damping (G), cmH_2O/mL	Control/ACS - FGR/NoACS	-1.5865	0.347	14.6	-4.571	**0.0020**
Tissue damping (G), cmH_2O/mL	Control/ACS - FGR/ACS	-0.2229	0.294	64.4	-0.759	0.8727
Tissue damping (G), cmH_2O/mL	FGR/NoACS - FGR/ACS	1.3636	0.364	15.1	3.743	**0.0094**
Tissue elastance (H), cmH_2O/mL	Control/NoACS - Control/ACS	0.490	0.983	19.2	0.498	0.9585
Tissue elastance (H), cmH_2O/mL	Control/NoACS - FGR/NoACS	-5.499	1.244	42.6	-4.422	**0.0004**
Tissue elastance (H), cmH_2O/mL	Control/NoACS - FGR/ACS	-0.478	1.071	19.5	-0.446	0.9696
Tissue elastance (H), cmH_2O/mL	Control/ACS - FGR/NoACS	-5.989	1.337	14.6	-4.480	**0.0024**
Tissue elastance (H), cmH_2O/mL	Control/ACS - FGR/ACS	-0.967	1.132	64.4	-0.854	0.8281
Tissue elastance (H), cmH_2O/mL	FGR/NoACS - FGR/ACS	5.022	1.403	15.1	3.579	**0.0129**
Hysteresis (A), $mL \cdot cmH_2O$	Control/NoACS - Control/ACS	-0.3022	0.0797	16.7	-3.790	**0.0074**
Hysteresis (A), $mL \cdot cmH_2O$	Control/NoACS - FGR/NoACS	0.2342	0.0795	65.8	2.946	**0.0225**
Hysteresis (A), $mL \cdot cmH_2O$	Control/NoACS - FGR/ACS	-0.0781	0.0859	18.6	-0.909	0.8004
Hysteresis (A), $mL \cdot cmH_2O$	Control/ACS - FGR/NoACS	0.5364	0.0993	25.6	5.401	**0.0001**
Hysteresis (A), $mL \cdot cmH_2O$	Control/ACS - FGR/ACS	0.2241	0.0732	62.9	3.064	**0.0166**
Hysteresis (A), $mL \cdot cmH_2O$	FGR/NoACS - FGR/ACS	-0.3123	0.1044	26.8	-2.992	**0.0283**

Note. Estimated differences, standard errors (SE), degrees of freedom (df), t ratios, and adjusted *P* values were derived from linear mixed-effects models including FGR status, ACS treatment, and their interaction as fixed effects and dam as a random intercept. Degrees of freedom were calculated using the Kenward-Roger method. Pairwise comparisons were based on estimated marginal means and adjusted for multiple comparisons using Tukey's method. The estimated difference represents the first group minus the second group listed in each contrast. Bold values indicate adjusted $P < 0.05$. Boundary (singular) fits occurred for respiratory system resistance, respiratory system elastance, tissue damping, and tissue elastance because the estimated dam-level random-intercept variance was zero. Inspiratory capacity and compliance outcomes were normalized to body weight. FGR, fetal growth restriction; ACS, antenatal corticosteroids; NoACS, no antenatal corticosteroids.

References

1. Derrick, M., et al., *Preterm fetal hypoxia-ischemia causes hypertonia and motor deficits in the neonatal rabbit: a model for human cerebral palsy?* J Neurosci, 2004. **24**(1): p. 24-34.
2. Georgiadis, P., et al., *Characterization of acute brain injuries and neurobehavioral profiles in a rabbit model of germinal matrix hemorrhage.* Stroke, 2008. **39**(12): p. 3378-88.
3. Kannan, S., et al., *Dendrimer-based postnatal therapy for neuroinflammation and cerebral palsy in a rabbit model.* Sci Transl Med, 2012. **4**(130): p. 130ra46.
